# Influence of Four-Week Glutamine Supplementation on Gut Microbiota Across a Training Period in Cross-Country Skiers

**DOI:** 10.3390/sports14090375

**Published:** 2026-09-01

**Authors:** Tung-Lin Lu, Kumpei Tanisawa, Zenya Fujita, Chikara Ozeki, Ena Yoshida, Shih-Hua Fang, Katsuhiko Suzuki

**Affiliations:** 1 Graduate School of Sport Sciences, Waseda University, Tokorozawa 359-1192, Saitama, Japan; chikka140630@akane.waseda.jp (C.O.); enayoshida9@asagi.waseda.jp (E.Y.); 2 Faculty of Sport Sciences, Waseda University, Tokorozawa 359-1192, Saitama, Japan; tanisawa@waseda.jp (K.T.); zenya.fujita@waseda.jp (Z.F.); katsu.suzu@waseda.jp (K.S.); 3 Institute of Athletics, National Taiwan University of Sport, Taichung 404401, Taiwan; shihhuafang@gmail.com

**Keywords:** L-glutamine, gut microbiota, endurance athletes, cross-country skiing, sports nutrition, 16S rRNA sequencing, gastrointestinal symptoms

## Abstract

The gut microbiota is increasingly recognized as an important factor related to intestinal homeostasis and athlete health, yet the effects of L-glutamine supplementation on gut microbiota composition in endurance athletes during training remain unclear. This randomized, double-blind, placebo-controlled crossover trial examined whether 4 weeks of L-glutamine supplementation influenced gut microbiota composition and gastrointestinal responses in 19 collegiate cross-country skiers during a training period. Stool samples were collected at baseline, week 2, and week 4 during each intervention period, and gut microbiota composition was analyzed by 16S rRNA gene sequencing targeting the V4 region. Shannon alpha diversity increased significantly over time, and Bray–Curtis beta diversity showed significant time-related changes, with no significant supplementation or interaction effects. At the genus level, L-glutamine supplementation was associated with a transient increase in *Faecalibacterium* and a decrease in *Collinsella* at week 2, though these changes were not sustained at week 4. Positive correlations among selected short-chain fatty acid-producing genera were also observed under the L-glutamine condition. Fecal calprotectin, gastrointestinal symptoms, stool form, and bowel movement frequency were not significantly affected by supplementation. These findings suggest that gut microbiota dynamics in collegiate cross-country skiers were characterized by time-dependent shifts across the intervention period, whereas L-glutamine supplementation was associated with transient genus-level shifts and microbial correlation patterns without adverse gastrointestinal responses.

## 1. Introduction

The gut microbiota is widely known as a critical contributor to host physiological processes, including metabolic regulation, immune responses, and maintenance of intestinal barrier function. In recent years, it has also attracted growing attention as a factor influencing athlete health and exercise performance [1].

Cross-country skiing represents a typical endurance sport involving prolonged training sessions, high energy demands, and repeated exposure to both environmental and physiological stressors. These conditions may influence gastrointestinal function and induce shifts in gut microbial composition throughout different phases of training [2]. It has been reported that endurance athletes exhibit distinctive gut microbial profiles compared with non-athletic individuals [3,4], typically characterized by greater microbial diversity and enrichment of short-chain fatty acid (SCFA)-producing bacteria. Endurance athletes show increased microbial diversity and higher abundance of taxa involved in fiber fermentation, such as Prevotella and Firmicutes-related bacteria [5,6], which are associated with enhanced metabolic efficiency and exercise adaptation. Despite these adaptations, endurance training may also impose physiological stress on the gastrointestinal system, potentially leading to compromised intestinal integrity and an increased incidence of gastrointestinal symptoms [7].

Given that the gut microbiota is highly responsive to dietary intake, nutritional strategies have been proposed as a means to regulate microbial composition and support athletic performance. Previous research has primarily focused on probiotic supplementation, which has shown potential benefits in modulating metabolic responses and exercise outcomes in endurance athletes [8,9]. However, relatively little is known about how specific amino acid supplementation, which is commonly used in athletes, affects the gut microbiota.

L-glutamine is commonly used as a nutritional supplement due to its role in supporting intestinal barrier function under conditions of physiological stress. Previous studies have shown that glutamine supplementation can attenuate exercise-induced increases in gastrointestinal permeability and intestinal damage markers, such as the lactulose-to-mannitol ratio and intestinal fatty acid–binding protein (I-FABP), in response to prolonged strenuous exercise [10]. In addition, glutamine supplementation has been reported to enhance mucosal immunity and reduce the incidence of upper respiratory tract infections in athletes, suggesting a protective role in maintaining intestinal and immune function during intensive training [11]. These findings indicate that glutamine may contribute to maintaining intestinal homeostasis under physiological stress, potentially through interactions with the gut microbiota.

Beyond these physiological effects, a 14-day intervention study demonstrated that oral supplementation with L-glutamine significantly altered gut microbial composition compared with an L-alanine control, including reductions in Firmicutes-associated genera and a decrease in the Firmicutes-to-Bacteroidetes ratio [12], suggesting that glutamine may have a capacity to modulate the gut environment through microbiota-mediated mechanisms. However, whether such microbiota-modulating effects occur in athletes during regular training remains unclear. Moreover, although glutamine supplementation has been reported to exert beneficial effects on intestinal function in endurance athletes, findings have been inconsistent.

Given the important roles of glutamine and the gut microbiota in maintaining intestinal homeostasis, it is unclear whether the effects of glutamine are mediated through alterations in microbial composition during training. Therefore, the present study aimed to examine the effects of L-glutamine supplementation on gut microbiota composition and gastrointestinal responses in cross-country skiers during a training period.

## 2. Materials and Methods

This study was approved by the Ethics Review Committee on Research of Human Subjects of Waseda University (approval No. 2024-342) and was conducted in accordance with the Declaration of Helsinki. All participants provided written informed consent prior to enrollment.

### 2.1. Study Design and Participants

Nineteen collegiate cross-country skiing athletes (male *n* = 9 and female *n* = 10) participated in a randomized, double-blind, placebo-controlled crossover study. Based on the Participant Classification Framework proposed by McKay et al. (2022), the cohort comprised athletes classified as Tier 3 (highly trained/national level) and Tier 4 (elite/international level), reflecting their structured training and competitive backgrounds [13]. Throughout the intervention periods, participants maintained structured team training routines and standardized dietary habits. The study was conducted during the general preparation phase of the season. Athletes participated in daily team training sessions lasting 1 to 2 h per day (typically 5 to 6 days per week, totaling approximately 6 to 12 h per week). The training protocol comprised running, strength and conditioning, roller skiing, pole walking, and high-intensity interval training. Daily meals were uniformly provided by the athlete dormitory, ensuring a high level of baseline dietary consistency across all participants throughout the study. Prior to enrollment, comprehensive baseline screening was conducted to assess medical history, medication use, dietary supplement intake, smoking habits, and alcohol consumption. Furthermore, participants refrained from consuming additional prebiotics, probiotics, fermented functional foods, or commercial amino acid supplements during the intervention periods.

### 2.2. Supplementation

Participants consumed L-glutamine or placebo (10 g/day; 5 g in the morning before breakfast and 5 g in the evening before dinner) for 4 weeks, with a 4-week washout period between conditions.

The selected dose (10 g/day) was based on previous studies demonstrating that glutamine supplementation in the range of approximately 0.1–0.3 g/kg or 5–10 g/day is commonly used in athletic populations and is considered safe and well tolerated [14,15].

The 4-week supplementation period was selected based on evidence that 2–4 weeks of glutamine interventions are sufficient to induce physiological responses in athletes [11,16].

### 2.3. Assessment

#### 2.3.1. Body Composition

Body fat percentage and fat-free mass were assessed before and after each intervention period using a multi-frequency bioelectrical impedance analyzer (InnerScan Dual RD-803L; TANITA Corporation, Tokyo, Japan).

#### 2.3.2. Questionnaires

Participants completed questionnaires regarding physical activity, smoking status, supplement intake, medication use, alcohol consumption, and general health status.

Gastrointestinal symptoms were assessed weekly using the Gastrointestinal Symptom Rating Scale (GSRS), a validated questionnaire widely used in previous clinical and exercise-related studies. The GSRS consists of 15 items rated on a 7-point Likert scale (1 = no discomfort to 7 = severe discomfort), and scores were calculated according to established methods, including five subdomains (reflux, abdominal pain, indigestion, diarrhea, and constipation), with the total GSRS score defined as the mean of all subscale scores [17].

Stool consistency was evaluated using the Bristol Stool Form Scale (BSFS), which has also been commonly applied in previous gastrointestinal and athlete-related studies [1,8]. Stool types were classified on a 7-point scale ranging from 1 (severe constipation) to 7 (severe diarrhea), with higher scores indicating looser stool consistency [18]. In addition, bowel movement frequency was recorded and defined as the number of bowel movements per week during the intervention period.

#### 2.3.3. Stool Sample Collection

Stool samples were collected at baseline, week 2, and week 4 during each intervention period within 3 days prior to each assessment session. Participants were instructed to collect stool samples immediately after defecation using designated collection kits according to the manufacturer’s instructions.

#### 2.3.4. Gut Microbiota Analysis

For gut microbiota analysis, fecal samples were collected using a commercially available fecal collection kit containing a Guanidine thiocyanate solution (TechnoSuruga Laboratory Co., Ltd., Shizuoka, Japan), allowing samples to be stored and transported at ambient temperature prior to microbiome analysis. Fecal bacterial DNA was extracted using the ISOSPIN Fecal DNA kit (NIPPON GENE Co., Ltd., Tokyo, Japan) according to the manufacturer’s protocol. DNA concentration was quantified using a Qubit 4 Fluorometer (Thermo Fisher Scientific, Waltham, MA, USA). The V4 region of the bacterial 16S rRNA gene was amplified using the 515F/806R primer set. Amplicon PCR and index PCR were performed using KAPA HiFi HotStart ReadyMix (Roche, Basel, Switzerland) following the Illumina 16S metagenomic library preparation workflow. PCR products were purified using AMPure XP beads, indexed with Nextera XT index primers, quantified, normalized, and pooled for sequencing.

Sequencing was performed using the Illumina iSeq 100 platform (Illumina, San Diego, CA, USA). Raw sequence data were processed using QIIME 2 software (version 2024.10). Paired-end reads were quality-filtered, denoised, merged, and checked for chimeras using the DADA2 plugin. Taxonomic assignment was performed using a pretrained SILVA 138.2 classifier targeting the V4 region of the 16S rRNA gene.

Alpha diversity was assessed using the Shannon diversity index. Beta diversity was calculated based on Bray–Curtis dissimilarity and visualized using principal coordinates analysis.

#### 2.3.5. Fecal Calprotectin Analysis

For fecal calprotectin analysis, samples were separately collected using a dedicated collection container, stored at −80 °C after collection, and maintained under frozen conditions until submission for analysis at a certified commercial laboratory (BML Inc., Tokyo, Japan).

### 2.4. Statistical Analysis

Statistical analyses were performed using SPSS software (version 25; IBM Corp., Armonk, NY, USA) and R software (version 4.3.3; R Foundation for Statistical Computing, Vienna, Austria). Data distribution was assessed using the Shapiro–Wilk test. Normally distributed variables are presented as the mean ± standard deviation, whereas non-normally distributed variables are presented as the median and interquartile range where appropriate. When normality assumptions were not satisfied, nonparametric tests or appropriate data transformation were applied.

For gut microbiota analysis, alpha diversity was evaluated using the Shannon diversity index. Differences in Shannon alpha diversity were analyzed using linear mixed-effects models with supplementation condition, timepoint, and their interaction as fixed effects and subject as a random effect. When required for exploratory within-condition comparisons, paired nonparametric tests were performed using the Wilcoxon signed-rank test. Beta diversity was assessed using Bray–Curtis dissimilarity and visualized by principal coordinates analysis. Differences in microbial community structure were tested using permutational multivariate analysis of variance (PERMANOVA). Homogeneity of multivariate dispersion was assessed using the betadisper test.

Relative abundances at the phylum and genus levels were analyzed using linear mixed-effects models. Supplementation condition, timepoint, and their interaction were included as fixed effects, and subject was included as a random effect. When significant main effects or interactions were detected, post hoc pairwise comparisons were performed with Bonferroni correction.

Gastrointestinal symptoms assessed using the Gastrointestinal Symptom Rating Scale (GSRS), stool consistency evaluated using the Bristol Stool Form Scale (BSFS), and bowel movement frequency were analyzed using linear mixed-effects models with supplementation condition, timepoint, and their interaction as fixed effects and subject as a random effect.

Fecal calprotectin concentrations were log-transformed prior to analysis. To account for the randomized crossover design, fecal calprotectin level was analyzed using a linear mixed-effects model including supplementation condition, timepoint, period, and relevant interaction terms as fixed effects, with subject included as a random effect.

Correlations among bacterial genera and between bacterial genera and fecal calprotectin concentrations were assessed using Spearman’s rank correlation analysis. *p*-values from correlation analyses were adjusted using the Benjamini–Hochberg false discovery rate (FDR) method. Statistical significance was set at *p* < 0.05.

## 3. Results

No adverse events, severe gastrointestinal discomfort, or side effects were reported during either the L-glutamine or placebo intervention periods. Baseline participant characteristics are presented in Table 1. No significant differences were observed in body composition parameters between supplementation conditions or across intervention periods, indicating that overall anthropometric status remained stable throughout the study. Because some fecal samples were unavailable at specific timepoints, the number of available microbiome samples differed slightly across timepoints. All available data were included in the analyses, and linear mixed-effects models were used to account for repeated measurements and unbalanced data caused by missing observations.

### 3.1. Gut Microbiota Diversity

**Figure 1:**
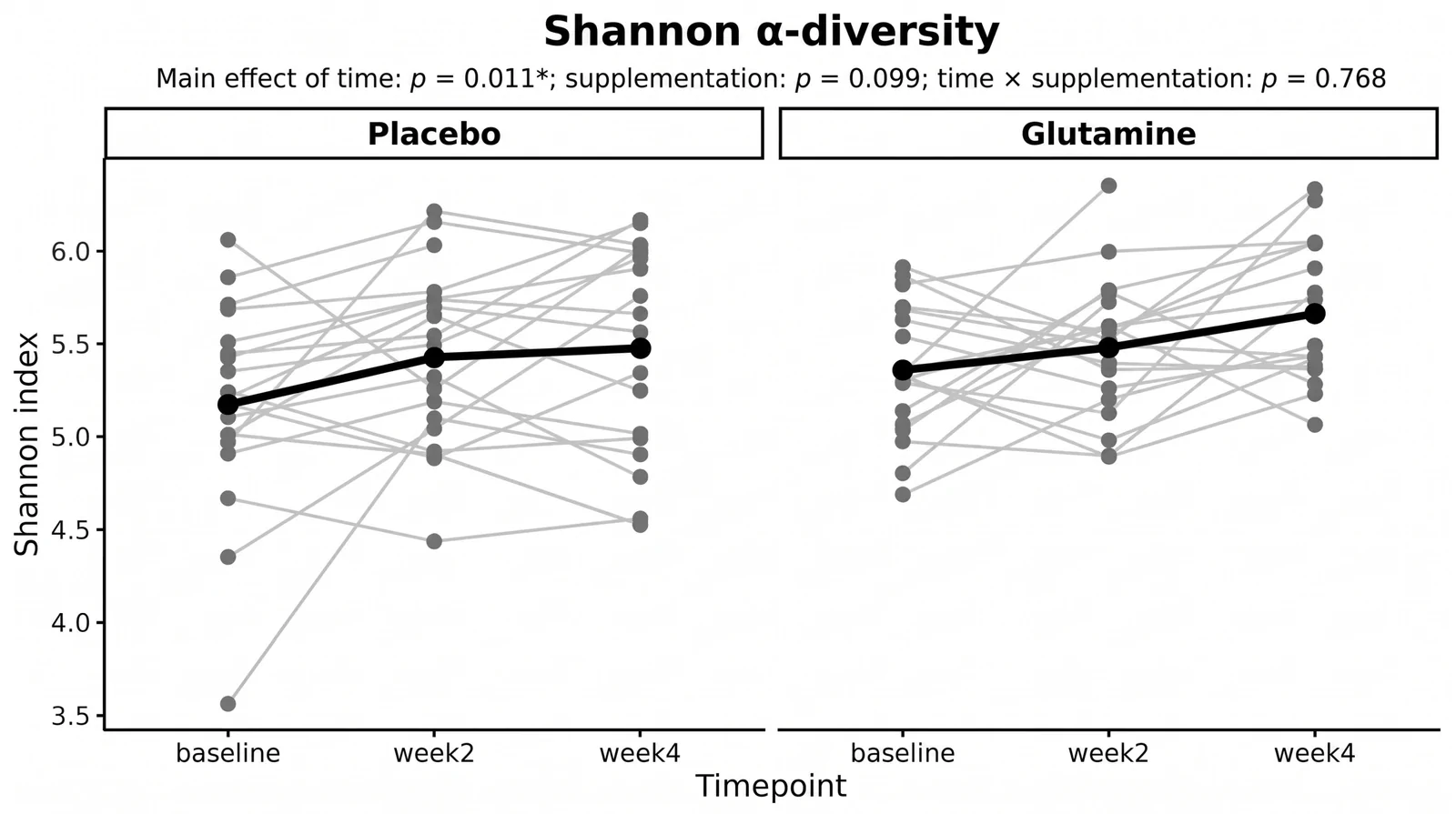
Changes in Shannon α-diversity during placebo and glutamine supplementation. Shannon α-diversity index at baseline, week 2, and week 4 during placebo and glutamine supplementation. Gray lines indicate individual trajectories, and black lines indicate mean values at each time point. Linear mixed-effects models showed a significant main effect of time (*p* = 0.011), whereas no significant main effect of supplementation or time × supplementation interaction was observed. * *p* < 0.05.

**Figure 2:**
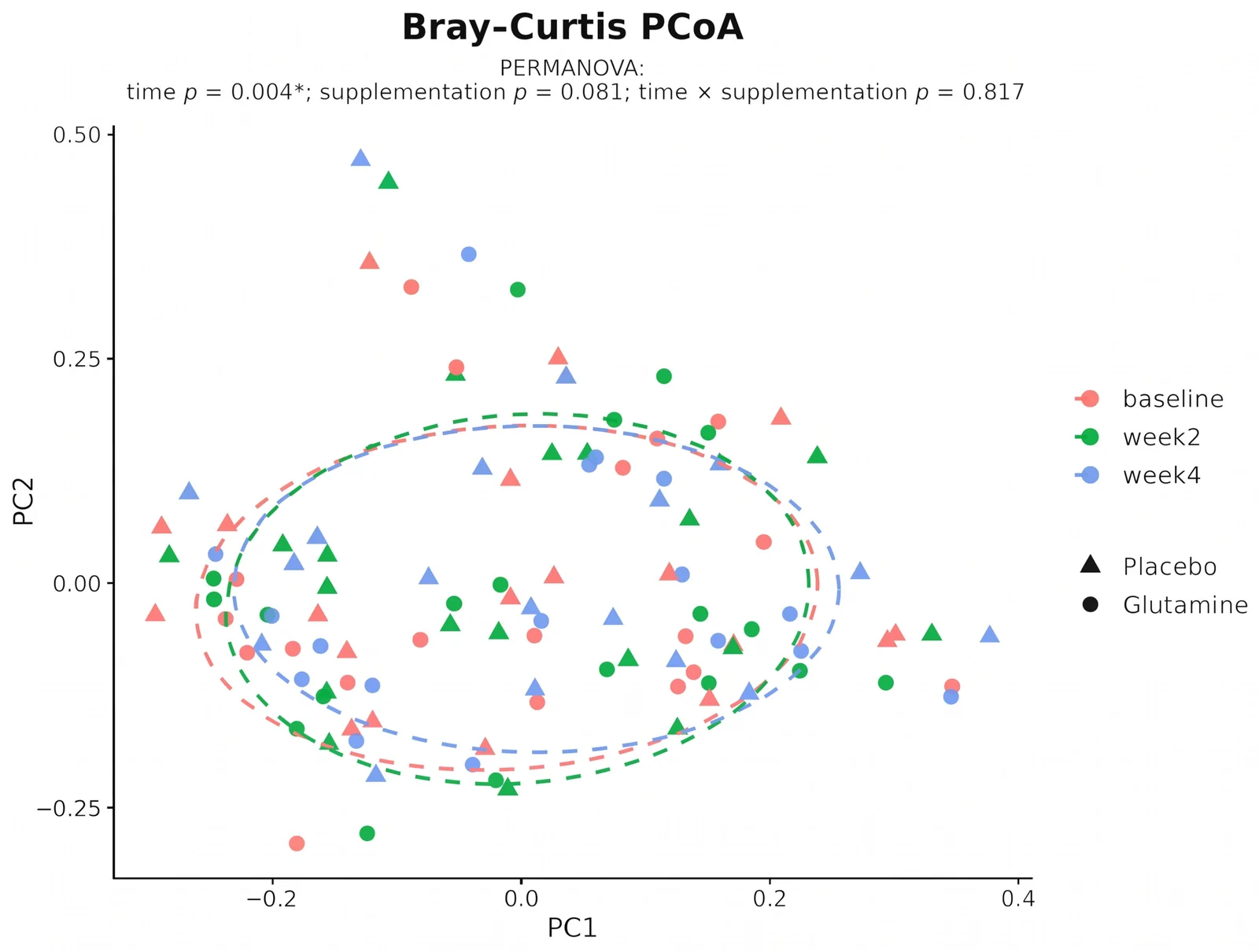
Bray–Curtis principal coordinates analysis during placebo and glutamine supplementation. Principal coordinates analysis (PCoA) plot based on Bray–Curtis dissimilarity. Points are colored by time point and shaped according to supplementation condition. PERMANOVA showed a significant effect of time point (*p* = 0.004), whereas supplementation condition and time × supplementation interaction were not significant. Homogeneity of multivariate dispersion was assessed using betadisper. * *p* < 0.05.

Shannon alpha diversity showed a significant main effect of time (*p* = 0.011), with values increasing from baseline to week 4, whereas no significant main effect of supplementation or time × supplementation interaction was observed (Figure 1). Similarly, beta diversity based on Bray–Curtis dissimilarity showed a significant effect of time (PERMANOVA, *p* = 0.004), while the effects of supplementation condition and the time × supplementation interaction were not significant (Figure 2). Homogeneity of multivariate dispersion was assessed using the betadisper test, indicating that the observed time-related differences reflected changes in community structure rather than differences in dispersion. These findings indicate that overall gut microbial diversity and community structure were primarily influenced by the training period rather than glutamine supplementation.

### 3.2. Gut Microbiota Composition

**Figure 3:**
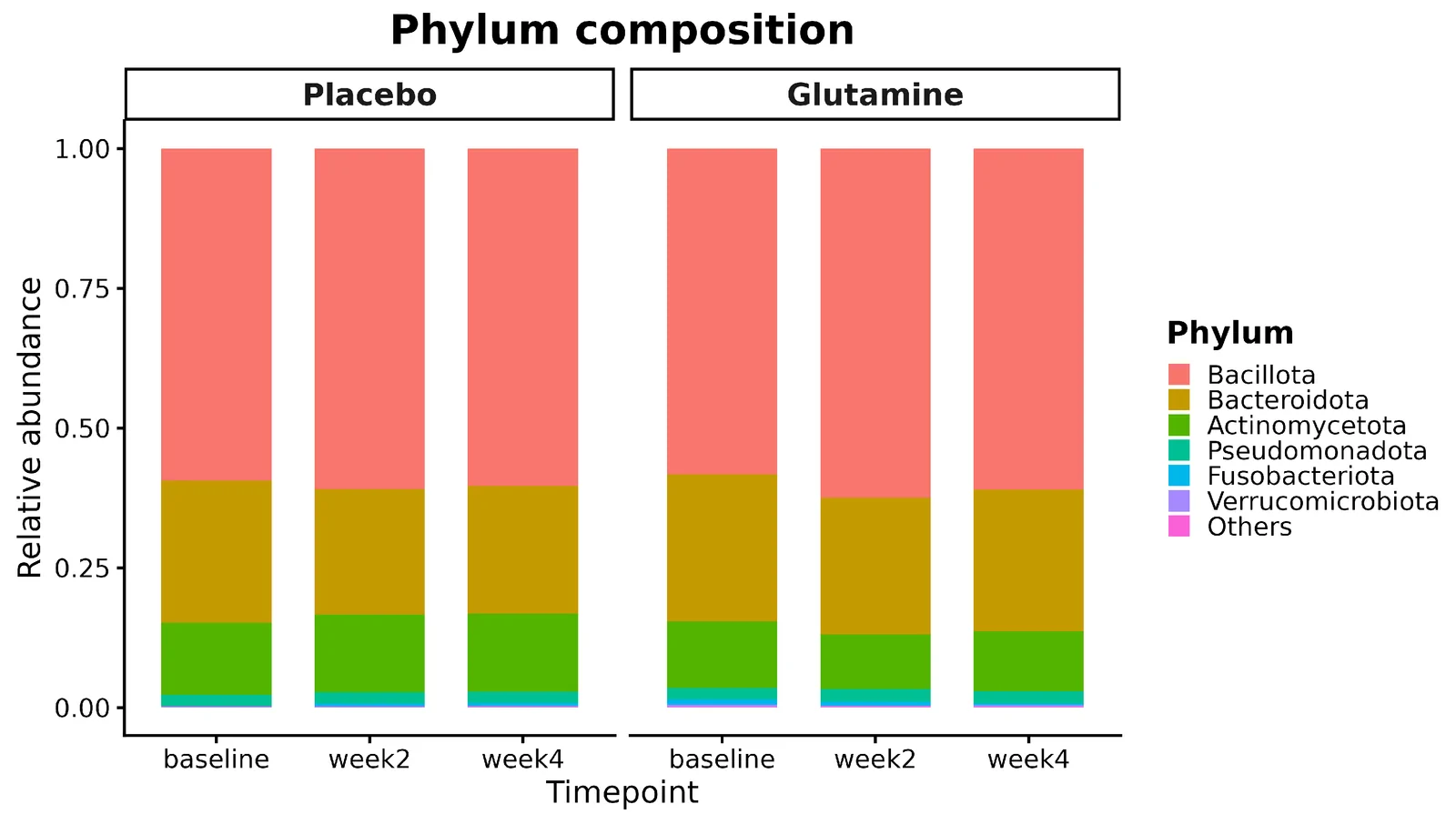
Phylum-level taxonomic composition during placebo and glutamine supplementation. Stacked bar plots show the mean relative abundance of major bacterial phyla at baseline, week 2, and week 4 during placebo and glutamine supplementation. Phyla with low relative abundance are grouped as “Others”. Linear mixed-effects models were used to examine the effects of supplementation condition, timepoint, and their interaction, with subject included as a random effect.

**Figure 4:**
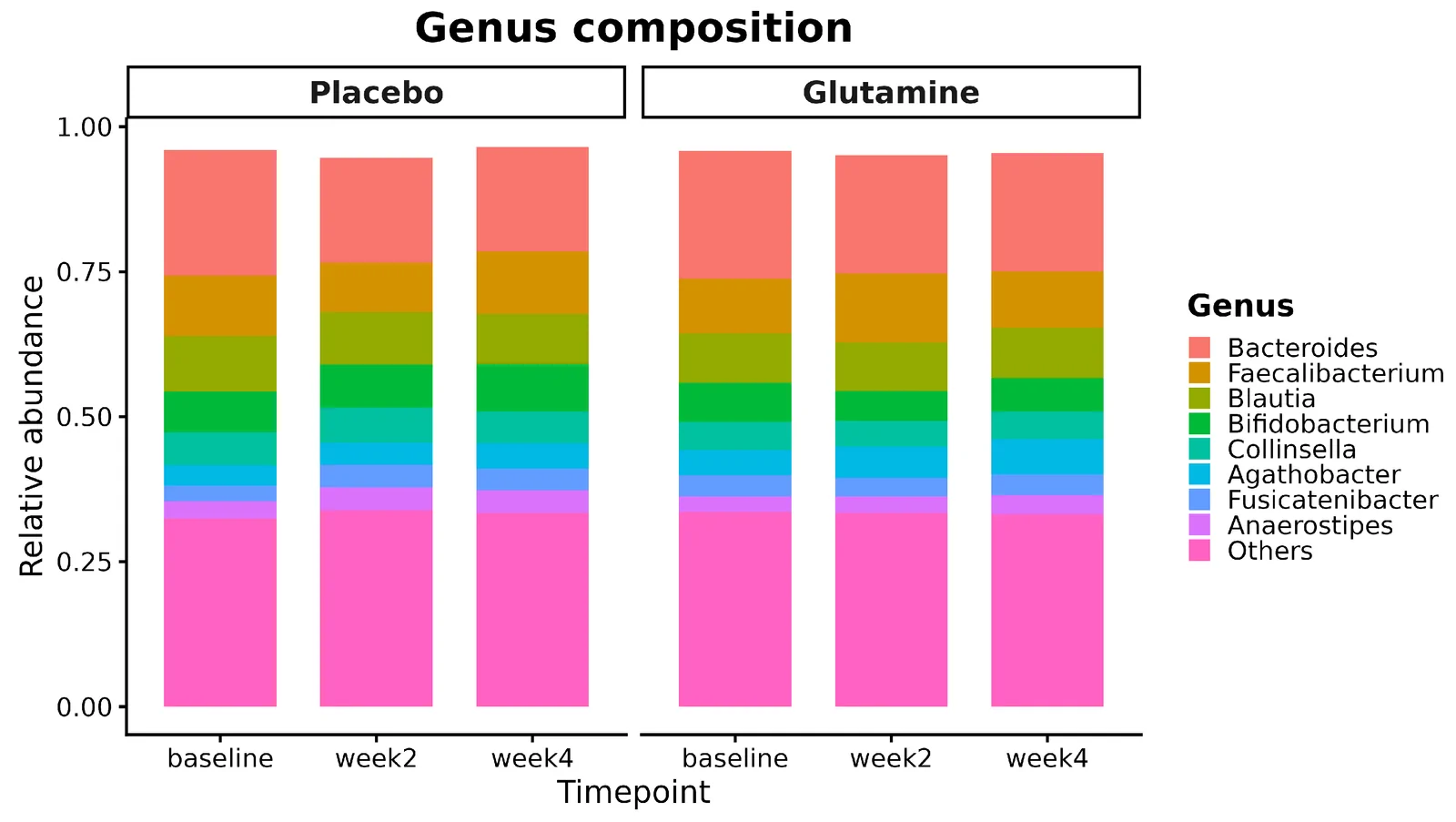
Genus-level taxonomic composition during placebo and glutamine supplementation. Stacked bar plots show the mean relative abundance of major bacterial genera at baseline, week 2, and week 4 during placebo and glutamine supplementation. Genera with low relative abundance are grouped as “Others”. Linear mixed-effects models were used to examine the effects of supplementation condition, timepoint, and their interaction, with subject included as a random effect.

**Figure A1:**
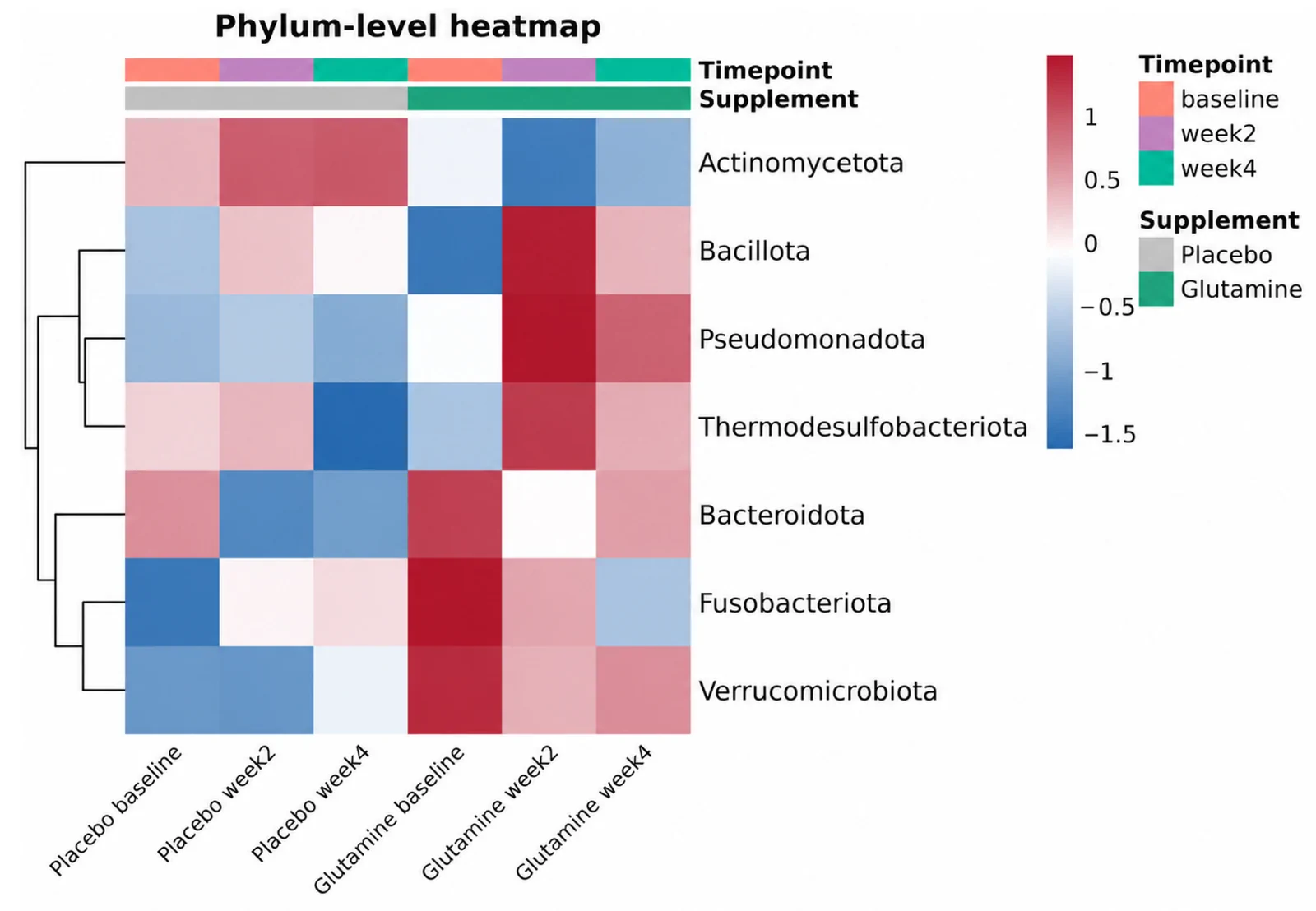
Heatmap of phylum-level taxonomic composition across timepoints and supplementation conditions. The heatmap shows standardized relative abundance of major bacterial phyla at baseline, week 2, and week 4 during placebo and glutamine supplementation. Rows represent bacterial phyla, and columns represent supplementation condition and timepoint. Color intensity indicates standardized relative abundance, with red indicating higher and blue indicating lower relative abundance.

**Figure A2:**
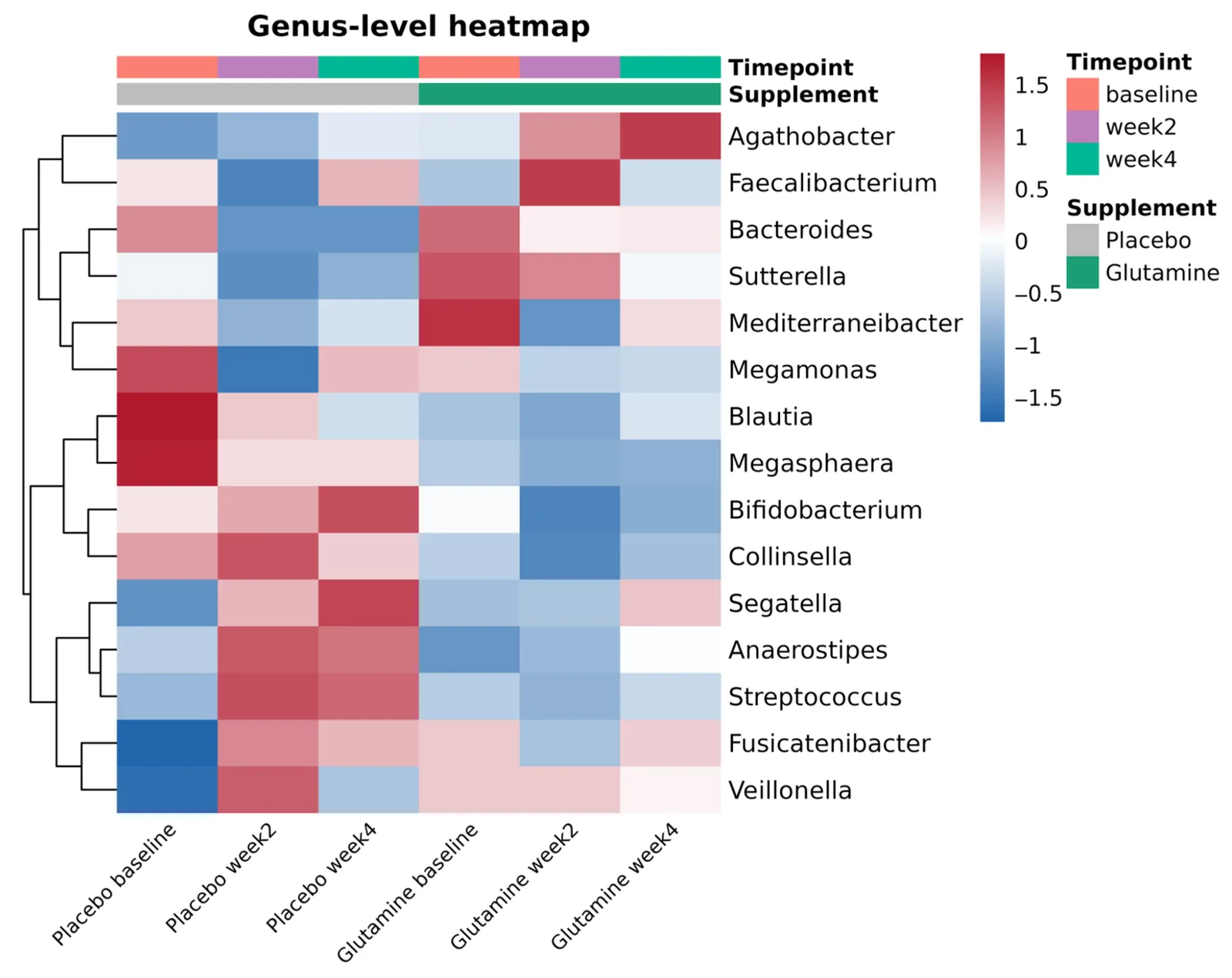
Heatmap of genus-level taxonomic composition across timepoints and supplementation conditions. The heatmap shows standardized relative abundance of major bacterial genera at baseline, week 2, and week 4 during placebo and glutamine supplementation. Rows represent bacterial genera, and columns represent supplementation condition and timepoint. Color intensity indicates standardized relative abundance, with red indicating higher and blue indicating lower relative abundance.

Phylum- and genus-level taxonomic compositions are shown in Figure 3 and Figure 4. Stacked bar plots present the mean relative abundance of major bacterial phyla and genera at baseline, week 2, and week 4 during placebo and glutamine supplementation. Heatmaps of standardized relative abundance are also presented to visualize taxonomic patterns across supplementation conditions and timepoints at the phylum and genus levels (Appendix A Figure A1 and Figure A2).

**Figure 5:**
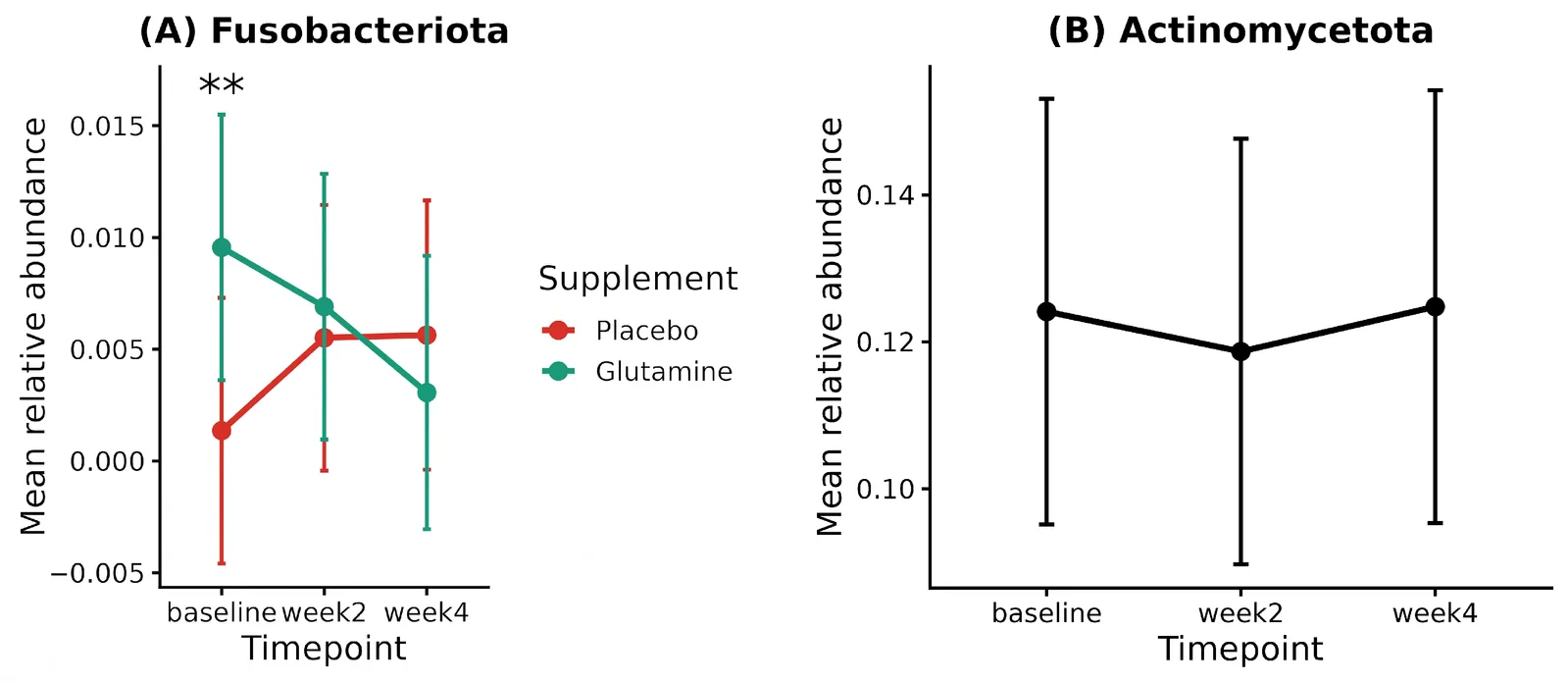
Phylum-level changes in Fusobacteriota and Actinomycetota during placebo and glutamine supplementation. Mean relative abundance of (**A**) Fusobacteriota and (**B**) Actinomycetota is shown at baseline, week 2, and week 4 during placebo and glutamine supplementation. Data are presented as mean ± SD. Linear mixed-effects models were used to examine the effects of supplementation condition, timepoint, and their interaction, with subject included as a random effect. A significant time × supplementation interaction was observed for Fusobacteriota (*p* = 0.005), which appeared to be mainly driven by baseline differences between supplementation conditions. Actinomycetota showed a significant main effect of time (*p* = 0.011). ** *p* < 0.01.

**Figure 6:**
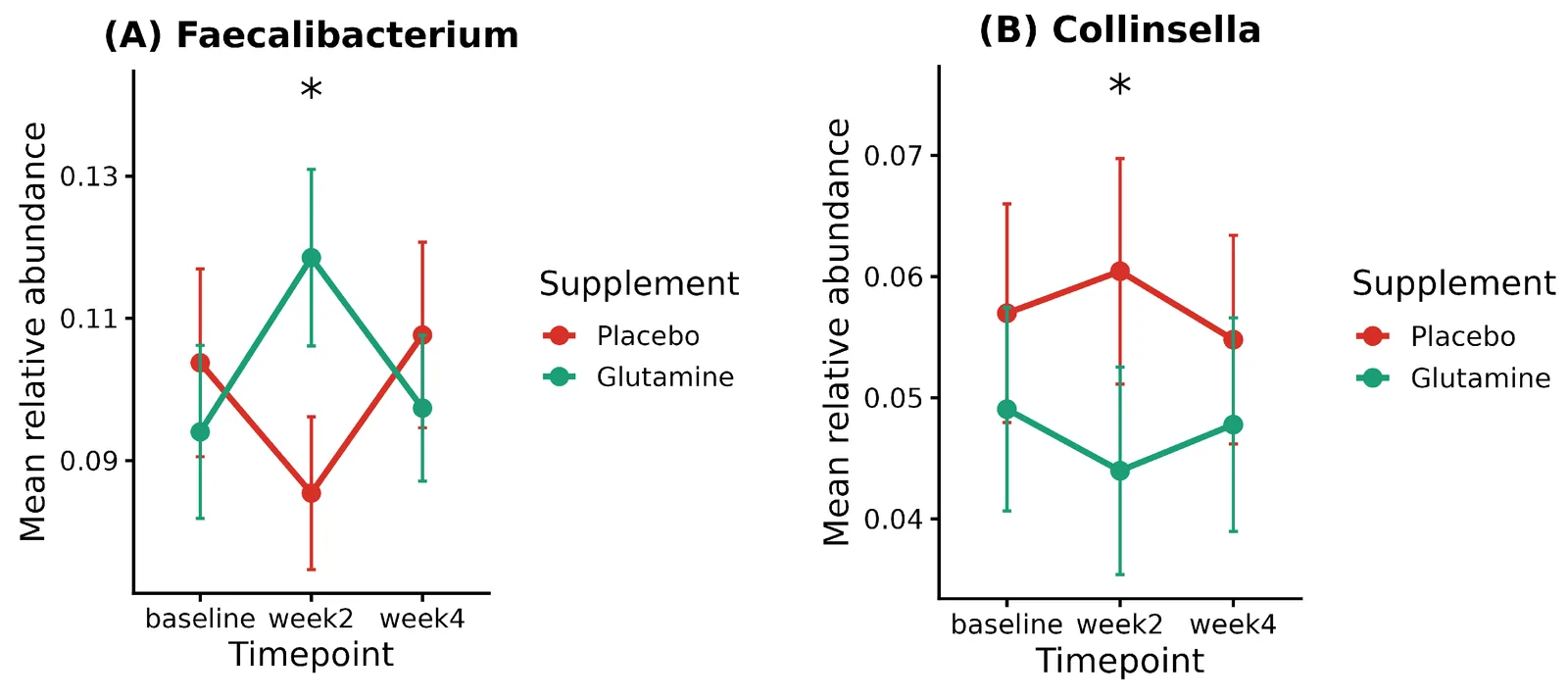
Genus-level changes in Faecalibacterium and Collinsella during placebo and glutamine supplementation. Mean relative abundance of (**A**) *Faecalibacterium* and (**B**) *Collinsella* is shown at baseline, week 2, and week 4 during placebo and glutamine supplementation. Data are presented as mean ± SD. Linear mixed-effects models were used to examine the effects of supplementation condition, timepoint, and their interaction, with subject included as a random effect. Significant time × supplementation interactions were observed for both genera. Post hoc comparisons showed higher relative abundance of *Faecalibacterium* (*p* = 0.010) and lower relative abundance of *Collinsella* (*p* = 0.020) at week 2 during glutamine supplementation. * *p* < 0.05.

At the phylum level, a significant time × supplementation interaction was observed for Fusobacteriota (*p* = 0.005, Figure 5A). However, post hoc comparisons suggested that this interaction was largely attributable to baseline differences between supplementation conditions, rather than a consistent supplementation-induced change during the intervention period. Linear mixed-effects models revealed a significant main effect of time for Actinomycetota (*p* = 0.011, Figure 5B), indicating a training period-related shift in phylum-level composition. At the genus level, significant time × supplementation interactions were observed for *Faecalibacterium* and *Collinsella*. Post hoc comparisons showed that, at week 2, the relative abundance of *Faecalibacterium* was higher during glutamine supplementation (*p* = 0.010, Figure 6A), whereas the relative abundance of *Collinsella* was lower during glutamine supplementation (*p* = 0.020, Figure 6B). These differences were not maintained at week 4, suggesting transient supplementation-specific shifts in genus-level composition.

Correlation patterns among SCFA-producing genera during supplementation.

**Figure 7:**
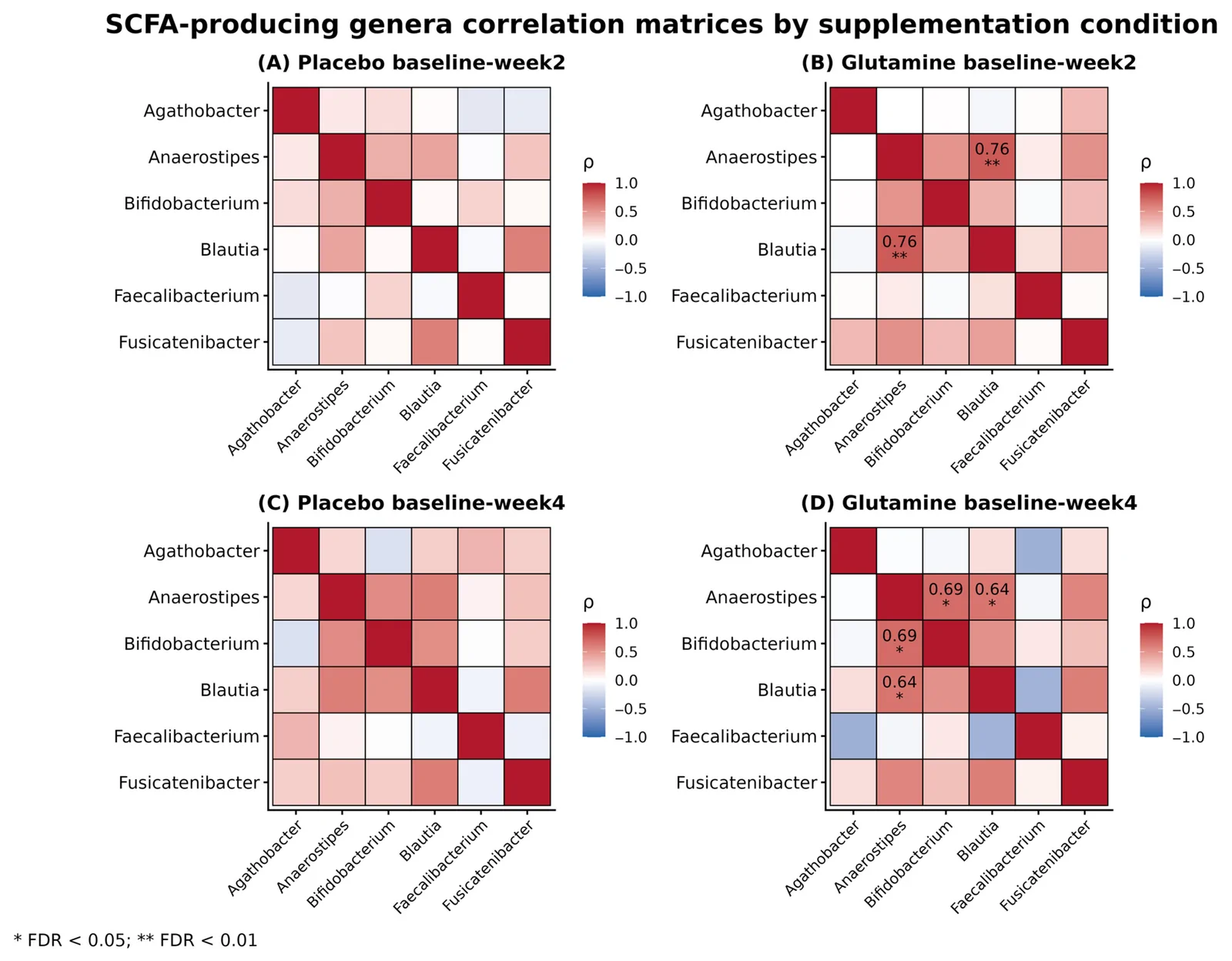
Correlation patterns among SCFA-producing genera according to supplementation conditions. Spearman correlation heatmaps showing associations among selected SCFA-producing genera based on changes in relative abundance from (**A**) placebo baseline to week 2, (**B**) glutamine baseline to week 2, (**C**) placebo baseline to week 4, and (**D**) glutamine baseline to week 4. From baseline to week 2, *Anaerostipes* was positively correlated with *Blautia* under the glutamine condition (ρ = 0.76, FDR = 0.002). From baseline to week 4, *Anaerostipes* was positively correlated with *Bifidobacterium* (ρ = 0.69, FDR = 0.032) and *Blautia* (ρ = 0.64, FDR = 0.039) under the glutamine condition. No significant correlations were observed under the placebo condition after FDR correction. *p*-values were adjusted using the Benjamini–Hochberg FDR method. * FDR < 0.05; ** FDR < 0.01.

Significant positive correlations among SCFA-producing genera were observed under the glutamine condition. *Anaerostipes* was positively correlated with *Blautia* from baseline to week 2 (ρ = 0.76, FDR = 0.002, Figure 7B). From baseline to week 4, *Anaerostipes* was positively correlated with both *Bifidobacterium* (ρ = 0.69, FDR = 0.032, Figure 7D) and *Blautia* (ρ = 0.64, FDR = 0.039, Figure 7D). No significant correlations were detected under the placebo condition after FDR correction.

### 3.3. Fecal Calprotectin, Gastrointestinal Symptoms and Stool Characteristics

Fecal calprotectin concentrations were not significantly affected by supplementation condition (*p* = 0.155), time (*p* = 0.829), or their interaction (*p* = 0.630), indicating that glutamine supplementation did not significantly influence intestinal inflammation during the intervention period. No significant associations between bacterial relative abundance and fecal calprotectin concentration were observed after FDR correction, although *Blautia* abundance showed a negative correlation tendency with fecal calprotectin concentration under the glutamine condition (ρ = −0.553, raw *p* = 0.026, FDR = 0.158).

Similarly, total GSRS score was not significantly affected by supplementation conditions, timepoint, or interaction. Among GSRS subscales, abdominal pain showed a significant main effect of time (*p* = 0.020), whereas reflux showed a trend over time (*p* = 0.082). No significant effects of supplementation, timepoint, or their interaction were observed for BSFS score or bowel movement frequency. Fecal calprotectin concentrations, gastrointestinal symptoms, and stool characteristics are summarized in Table 2.

## 4. Discussion

L-glutamine supplementation did not induce major overall changes in gut microbial diversity or community structure in collegiate cross-country skiers during a training period. However, it was associated with transient genus-level changes, including higher relative abundance of *Faecalibacterium* and lower relative abundance of *Collinsella* at week 2, as well as distinct correlation patterns among SCFA-producing genera.

Previous studies show that exercise can modify the gut microbial environment in athletes. Tabone et al. reported that even an acute moderate-intensity exercise bout altered selected bacterial taxa and serum/fecal metabolic profiles in cross-country endurance athletes, indicating that exercise itself can induce detectable changes in gut microbiota and related metabolism [2]. Toda et al. investigated Japanese male handball players across in-season and off-season periods and observed a significant longitudinal change in the Shannon alpha diversity in athletes, whereas no such change was observed in non-athletic subjects. They also reported that alpha diversity was higher during the in-season than during the off-season, supporting the concept that gut microbial diversity in athletes can fluctuate according to seasonal or training-related status [19]. Consistent with previous observations, our findings demonstrated time-dependent shifts in microbial diversity across the intervention period. Shannon alpha diversity increased over time, and Bray–Curtis beta diversity showed a significant time-related shift, whereas no significant supplementation or time × supplementation interaction effects were observed.

Karl et al. demonstrated that a 4-day military cross-country ski-march, used as a model of prolonged physiological stress, increased intestinal permeability and altered intestinal microbiota composition and metabolism [20], indicating that an increase in alpha diversity under substantial physiological stress should not necessarily be interpreted as beneficial; rather, it may reflect a stress-related ecological response of the gut microbiota. In the present study, however, fecal calprotectin concentrations and gastrointestinal symptoms were not significantly altered, suggesting that the observed diversity changes did not indicate overt intestinal inflammation or adverse gastrointestinal responses. Taken together, these findings suggest that the diversity changes observed in this study may reflect time-dependent microbial dynamics across the intervention period rather than being a direct effect of L-glutamine supplementation.

At the phylum level, Actinomycetota showed a significant main effect of time, further supporting the interpretation that taxonomic composition changed along with the progression of the training period. This time-dependent shift may be biologically relevant because Actinomycetota includes several taxa involved in carbohydrate metabolism, including *Bifidobacterium*, a genus known to participate in carbohydrate fermentation and acetate production, thereby contributing to intestinal metabolic homeostasis [21,22]. Karl et al. reported that pre-stress Actinomycetota relative abundance was associated with changes in intestinal permeability during prolonged physiological stress, suggesting a potential role of this phylum in intestinal barrier responses to stress [20]. Previous evidence also shows that training status and periodization can influence gut microbial composition in athletes. Akazawa et al. reported that the gut microbiota composition of Japanese elite athletes differed across training periodization phases, and that longitudinal changes in specific genera, including *Bacteroides*, *Blautia*, and *Bifidobacterium*, were associated with changes in physical fitness [1]. Therefore, the observed time-related change in Actinomycetota may reflect training period-related microbial adaptation rather than a glutamine-specific response.

Although Fusobacteriota showed a significant time × supplementation interaction in the present study, post hoc comparisons indicated that this interaction was largely attributable to baseline differences between supplementation conditions. Therefore, this result should be interpreted cautiously and does not provide strong evidence for a consistent supplementation-induced alteration in Fusobacteriota during the intervention.

At the genus level, glutamine supplementation was associated with a transiently higher relative abundance of *Faecalibacterium* at week 2 compared with placebo, whereas this difference was not maintained at week 4. *Faecalibacterium* is one of the major commensal genera in the human gut and has been widely associated with intestinal health, butyrate production, and anti-inflammatory activity [23]. *Faecalibacterium prausnitzii* has been described as an anti-inflammatory commensal bacterium, and reduced abundance of this species has been reported in inflammatory intestinal diseases such as Crohn’s disease [24,25]. Therefore, the higher relative abundance of *Faecalibacterium* observed at week 2 may indicate a short-term shift toward a microbial profile associated with butyrate production and an anti-inflammatory intestinal environment. This transient response may be partly related to the indirect effects of glutamine on the intestinal microenvironment. Glutamine is an important energy substrate for intestinal epithelial and immune cells and has been implicated in the maintenance of intestinal barrier function, regulation of tight junction integrity, and modulation of inflammatory responses [26]. Therefore, glutamine may not directly stimulate *Faecalibacterium* as a classical prebiotic substrate, but it may transiently create a gut environment that favors butyrate-associated commensal bacteria. A previous human pilot study using a synbiotic formulation containing Bacillus subtilis DSM 32315 and L-alanyl-L-glutamine reported increased fecal butyrate levels and butyrate-producing taxa, including *Faecalibacterium prausnitzii*, in healthy adults [27]. However, the higher relative abundance of *Faecalibacterium* during glutamine supplementation was no longer evident at week 4, suggesting an early and transient genus-level response rather than a sustained increase. In highly trained athletes, gut microbiota responses may be influenced by multiple factors, including training load, dietary intake, host metabolism, recovery status, and inter-individual variability [28,29], which may partly explain why the initial difference was not maintained.

In contrast to *Faecalibacterium*, the relative abundance of *Collinsella* was lower at week 2 during glutamine supplementation, and this difference was also not maintained at week 4. This opposite pattern between *Faecalibacterium* and *Collinsella* may reflect a transient shift in genus-level microbial ecology during the early phase of glutamine supplementation. *Collinsella* is a member of the Actinomycetota and has been implicated in carbohydrate utilization and host lipid-related metabolic profiles [30,31]. However, unlike *Faecalibacterium*, which is generally regarded as a butyrate-producing commensal genus, the biological interpretation of *Collinsella* is context-dependent [32]. Higher abundance of *Collinsella* has been reported in some metabolic or inflammatory conditions, including symptomatic atherosclerosis, nonalcoholic steatohepatitis, and established rheumatoid arthritis [30,32,33]. In addition, *Collinsella aerofaciens* may increase gut permeability and promote IL-17-related inflammatory responses, supporting a potential role of *Collinsella* in epithelial barrier and immune regulation in inflammatory disease contexts [34]. In contrast, Furber et al. reported that high-carbohydrate dietary periodization in highly trained endurance runners improved time-trial performance and was associated with expansion of *Ruminococcus* and *Collinsella*, indicating that *Collinsella* may also increase under performance-supportive nutritional conditions in athletes [4]. Therefore, the lower relative abundance of *Collinsella* alone might not be interpreted as direct evidence of improved gut health. However, in the present study, together with the transient increase in *Faecalibacterium*, the pattern may have resulted from indirect effects of glutamine on the intestinal microenvironment, which may indicate a short-term shift toward a microbial profile characterized by greater butyrate-associated commensal representation and lower abundance of taxa linked to metabolic-inflammatory conditions.

In addition to the transient genus-level changes, glutamine supplementation was associated with distinct correlation patterns among SCFA-related genera. Significant positive correlations were observed among *Anaerostipes*, *Blautia*, and *Bifidobacterium* under the glutamine condition, whereas no significant correlations were detected under the placebo condition after FDR correction. These genera have complementary roles in SCFA-related metabolism. *Bifidobacterium* produces acetate and lactate through carbohydrate fermentation, which can be used by other anaerobic bacteria through metabolic cross-feeding [21]. *Anaerostipes* is a lactate- and acetate-utilizing butyrate-producing genus [35], and its cross-feeding between *Bifidobacterium* and butyrate-producing colon bacteria has been proposed as an important mechanism supporting butyrate formation in the gut. *Blautia* is also an SCFA-associated commensal genus [36], and recent experimental evidence suggests that *Blautia*-derived SCFAs may contribute to maintaining colonic mucus function [37]. Therefore, the positive correlations observed in the present study may reflect coordinated changes among metabolically connected commensal bacteria involved in SCFA-related microbial ecology. The fact that these associations were observed only during glutamine supplementation may indicate that glutamine may have altered the local intestinal environment in a way that allowed SCFA-related taxa to fluctuate more coordinately. This interpretation is consistent with the genus-level findings showing a transient increase in *Faecalibacterium* in week 2 and suggests that glutamine may affect the ecological organization of functionally related bacteria more than overall taxonomic composition.

In endurance athletes, this finding may be relevant because SCFAs are involved in host energy metabolism, immune regulation, and intestinal barrier function, which are important during repeated training stress. Athlete microbiome studies have emphasized that gut microbial composition and function are shaped by both training and diet, and that SCFA-associated taxa may be related to performance and recovery. Nevertheless, direct evidence linking coordinated changes among *Bifidobacterium*, *Anaerostipes* and *Blautia* to exercise performance or gut-related outcomes in athletes remains limited. Therefore, the present correlation results should be regarded as exploratory and future studies should include fecal SCFA measurements and performance outcomes to determine whether these microbial co-occurrence patterns reflect functional adaptations during endurance training.

This study has several limitations. The sample size was relatively small, although the randomized crossover design allowed each participant to serve as their own control and helped reduce inter-individual variability. Gut microbiota composition was assessed using 16S rRNA gene sequencing, which provides taxonomic information but does not directly evaluate microbial functional capacity. Since fecal SCFA concentrations, metagenomic functional profiles, and metabolomic data were not measured, it remains unclear whether the observed correlations among SCFA-producing genera reflected actual functional metabolic changes. These interpretations must be presented strictly as speculative hypotheses rather than direct evidence of altered metabolic output. Therefore, the present correlation results should be regarded as exploratory.

The 4-week supplementation period in the present study represents a short-to-medium-term trial. Although evidence suggests that a 2-to-4-week L-glutamine intervention is sufficient to elicit early mucosal immune and physiological responses in athletic populations [11,16], it may be insufficient to induce sustained, long-term structural remodeling of the gut microbiota. Therefore, prolonged supplementation strategies such as across multiple training macrocycles or an entire competitive season (e.g., >8–12 weeks) are needed to determine whether extended L-glutamine administration fosters persistent microbial community shifts and long-term gastrointestinal adaptations under chronic training stress.

Although participants maintained their usual training schedules during the intervention periods, detailed changes in training load, dietary intake, and recovery status were not fully controlled. These factors may have contributed to the time-related changes in gut microbial diversity and community structure. In addition, some fecal samples were unavailable at specific time points, resulting in slightly unbalanced data. Linear mixed-effects models were used to account for repeated measurements and missing observations; however, missing samples may still have reduced statistical power. Fecal calprotectin was used as an intestinal inflammation marker, but intestinal permeability and epithelial damage markers, such as lactulose-to-mannitol ratio or intestinal fatty acid–binding protein, were not assessed. Therefore, the present study could not determine whether L-glutamine influenced intestinal barrier function directly.

Additionally, biological sex was not evaluated as an independent variable in the present study due to sample size constraints. Emerging evidence suggests that host biological sex and sex hormones can interact with the gut microbiota, leading to baseline differences in microbial diversity, phylum level composition, and exercise-induced adaptations [38,39]. Future studies with larger, sex-stratified athletic cohorts are required to clarify whether L-glutamine supplementation exerts sex-specific effects on gut microbiota dynamics and intestinal homeostasis during intensive training.

Despite these limitations, this study provides novel evidence regarding the effects of L-glutamine supplementation on gut microbiota composition in elite collegiate cross-country skiers during a training period.

Furthermore, it is worth noting that future nutritional strategies could explore the potential synergistic effects of combining L-glutamine with other anti-inflammatory or immunomodulatory supplements. For instance, combining L-glutamine with prebiotics, probiotics, or anti-inflammatory agents such as omega-3 fatty acids, which have been shown to modulate exercise-induced inflammation and enhance post-exercise recovery in athletes [5,27,40], could exert complementary benefits on mucosal/systemic inflammation and supporting microbial homeostasis [27,40]. Investigating such multi-ingredient strategies represents a promising direction for optimizing gastrointestinal health and physical recovery in endurance athletes.

Future studies with larger, sex-stratified cohorts and controlled dietary and training records are needed to validate and extend these findings. Incorporating prolonged supplementation durations alongside co-supplementation strategies will provide deeper insights into sustained microbial adaptations. Integrating metagenomic sequencing, fecal SCFA quantification, and direct intestinal permeability markers across diverse sports disciplines will help clarify whether the transient microbial shifts and SCFA-related correlation patterns observed in this study translate into functional relevance for athlete health and performance under specific physical demands.

## 5. Conclusions

In conclusion, gut microbiota diversity and community structure in collegiate cross-country skiers were primarily characterized by time-dependent shifts across the 4-week training period. L-glutamine supplementation did not alter microbial community diversity or structure, nor did it significantly impact gastrointestinal symptoms, stool characteristics, or fecal calprotectin levels. L-glutamine administration was associated only with transient genus-level shifts at week 2—specifically an increase in *Faecalibacterium* and a decrease in *Collinsella*—which were not sustained at week 4. In addition, while positive correlations among putative SCFA-producing genera were observed under the L-glutamine condition, these exploratory co-occurrence patterns suggest that L-glutamine may subtly influence local microbial ecological dynamics rather than broadly remodeling gut microbiota composition.

## Figures and Tables

**Table 1:** Baseline participant characteristics by supplementation condition.

Variable	Placebo Condition (n = 19)	Glutamine Condition (n = 19)
Age, years	20.1 ± 2.0	20.7 ± 1.4
Height, cm	167.1 ± 9.7	167.0 ± 9.8
Body weight, kg	61.8 ± 7.1	61.7 ± 7.0
Fat-free mass, kg	50.8 ± 9.7	51.1 ± 10.0
Body fat, %	18.4 ± 7.7	18.6 ± 8.6
BMI, kg/m^2^	22.1 ± 1.0	22.0 ± 1.1

Values are presented as mean ± SD. Body composition was measured before each supplementation period. BMI, body mass index.

**Table 2:** Fecal calprotectin concentration, gastrointestinal symptoms, and stool characteristics during placebo and glutamine supplementation. (**A**). Fecal calprotectin concentration across baseline, week 2, and week 4. (**B**). Gastrointestinal symptoms and stool characteristics.

(**A**)							
Variable	Condition	Baseline	Week 2	Week 4	Supplementation *p*	Timepoint *p*	Supplementation × Timepoint *p*
Fecal calprotectin, μg/g	Placebo	18.9 [8.2–54.9]	17.9 [9.7–44.5]	21.9 [16.5–45.3]	0.155	0.829	0.630
	Glutamine	29.4 [13.8–69.3]	15.8 [11.7–66.8]	22.9 [12.7–48.6]			
(**B**)							
Variable	Placebo	Glutamine	Supplementation *p*		Timepoint *p*	Supplementation × timepoint *p*	
Total GSRS score	1.22 ± 0.45	1.22 ± 0.41	0.758		0.136	0.498	
Indigestion	1.17 ± 0.41	1.23 ± 0.48	0.630		0.539	0.611	
Abdominal pain	1.21 ± 0.65	1.21 ± 0.67	0.977		0.020 *	0.175	
Reflux	1.06 ± 0.30	1.16 ± 0.50	0.249		0.082	0.325	
Diarrhea	1.35 ± 0.89	1.22 ± 0.53	0.376		0.283	0.823	
Constipation	1.30 ± 0.76	1.27 ± 0.60	0.787		0.385	0.872	
BSFS score	3.94 ± 0.98	3.80 ± 1.09	0.248		0.897	0.267	
Bowel movement frequency	8.69 ± 2.98	8.52 ± 2.88	0.802		0.848	0.375	

Values are presented as mean ± SD, except for fecal calprotectin, which is presented as median [IQR]. For fecal calprotectin, available observations were n = 19 at baseline and week 2 for both conditions, n = 18 at placebo week 4, and n = 17 at glutamine week 4 because of missing samples. *p*-values were derived from linear mixed-effects models. Fecal calprotectin concentrations were log-transformed prior to statistical analysis. GSRS, Gastrointestinal Symptom Rating Scale; BSFS, Bristol Stool Form Scale; IQR, interquartile range. * *p* < 0.05.

## Data Availability

The data presented in this study are available from the corresponding author upon reasonable request. The data are not publicly available due to privacy and ethical restrictions related to human participant data.

## Abbreviations

The following abbreviations are used in this manuscript:

BMI	body mass index
BSFS	Bristol Stool Form Scale
FDR	false discovery rate
GSRS	Gastrointestinal Symptom Rating Scale
LMM	linear mixed-effects model
PCoA	principal coordinate analysis
PERMANOVA	permutational multivariate analysis of variance
SCFA	short-chain fatty acid
VO_2_max	maximal oxygen uptake
